# Whole-Exome Sequencing in Searching for New Variants Associated With the Development of Parkinson’s Disease

**DOI:** 10.3389/fnagi.2018.00136

**Published:** 2018-05-15

**Authors:** Marina V. Shulskaya, Anelya Kh. Alieva, Ivan N. Vlasov, Vladimir V. Zyrin, Ekaterina Yu. Fedotova, Natalia Yu. Abramycheva, Tatiana S. Usenko, Andrei F. Yakimovsky, Anton K. Emelyanov, Sofya N. Pchelina, Sergei N. Illarioshkin, Petr A. Slominsky, Maria I. Shadrina

**Affiliations:** ^1^Laboratory of Molecular Genetics of Hereditary Diseases, Institute of Molecular Genetics, Russian Academy of Sciences (RAS), Moscow, Russia; ^2^Federal State Scientific Institution, Scientific Center of Neurology, Russian Academy of Sciences (RAS), Moscow, Russia; ^3^The Petersburg Nuclear Physics Institute of the National Research Center, Kurchatov Institute, Russian Academy of Sciences (RAS), Gatchina, Russia; ^4^Federal State Budgetary Educational Institution of Higher Education, Pavlov First Saint Petersburg State Medical University, Saint Petersburg, Russia

**Keywords:** Parkinson’s disease, whole-exome sequencing, NGS, variant, mutation

## Abstract

**Background**: Parkinson’s disease (PD) is a complex disease with its monogenic forms accounting for less than 10% of all cases. Whole-exome sequencing (WES) technology has been used successfully to find mutations in large families. However, because of the late onset of the disease, only small families and unrelated patients are usually available. WES conducted in such cases yields in a large number of candidate variants. There are currently a number of imperfect software tools that allow the pathogenicity of variants to be evaluated.

**Objectives**: We analyzed 48 unrelated patients with an alleged autosomal dominant familial form of PD using WES and developed a strategy for selecting potential pathogenetically significant variants using almost all available bioinformatics resources for the analysis of exonic areas.

**Methods**: DNA sequencing of 48 patients with excluded frequent mutations was performed using an Illumina HiSeq 2500 platform. The possible pathogenetic significance of identified variants and their involvement in the pathogenesis of PD was assessed using SNP and Variation Suite (SVS), Combined Annotation Dependent Depletion (CADD) and Rare Exome Variant Ensemble Learner (REVEL) software. Functional evaluation was performed using the Pathway Studio database.

**Results**: A significant reduction in the search range from 7082 to 25 variants in 23 genes associated with PD or neuronal function was achieved. Eight (*FXN*, *MFN2*, *MYOC*, *NPC1*, *PSEN1*, *RET*, *SCN3A* and* SPG7*) were the most significant.

**Conclusions**: The multistep approach developed made it possible to conduct an effective search for potential pathogenetically significant variants, presumably involved in the pathogenesis of PD. The data obtained need to be further verified experimentally.

## Introduction

Parkinson’s disease (PD) is one of the most common human neurodegenerative disorders and belongs to the category of multifactorial diseases. In most cases, the disease is sporadic and is associated with a complex interaction of genetic and environmental factors. However, families with monogenic inheritance of PD have been identified, which has made it possible to reveal a number of genes, i.e., mutations that lead to the development of hereditary forms of the disease (Singleton and Hardy, 2016). Analysis of all available studies of the familial form of PD indicates that the fraction of monogenic forms does not account for more than 10% of all cases, whereas the expected number of cases attributable from a genetic viewpoint is 27%–40% (Hamza and Payami, 2010; Keller et al., 2012). In this regard, it is expedient to continue searching and identifying genes and mutations that lead to the development of monogenic forms of PD.

High throughput next-generation sequencing (NGS) has significantly accelerated the search process. The technology of whole-exome sequencing (WES) has allowed expansion of the spectrum of mutations in genes that were previously associated with PD pathogenesis (Gorostidi et al., 2016). However, the most interesting application of the technology is the search for new genes that are the genetic cause of various diseases. To do this, as a rule, large families with several generations in the pedigree are studied, and potential pathogenically significant variants are analyzed using the method of cosegregation within such a family. Over the past few years, the use of this approach in combination with NGS technologies has made it possible to identify a number of new genes involved in the pathogenesis of PD. These include *VPS35*, *CHCHD2* and *DNAJC13*, associated with the development of the familial autosomal dominant form of the PD (Vilariño-Güell et al., 2011; Zimprich et al., 2011), *DNAJC6, SYNJ1, VPS13C* and *PTRHD1* involved in the development of autosomal recessive form of PD (Edvardson et al., 2012; Lesage et al., 2016; Jansen et al., 2017; Khodadadi et al., 2017), and *RAB39B* with X-linked inheritance (Wilson et al., 2014).

However, PD is characterized by a late onset; therefore, large families consisting of several generations are extremely rare and, in most cases, one has to analyze small families and single unrelated patients. WES is known to identify up to 20,000 single-nucleotide variants (SNVs) in one analyzed sample (Bamshad et al., 2011), which leads to the emergence of a huge number of candidate variants and the need to develop a strategy for selecting a narrow range of potentially pathogenic variants. Currently, a number of imperfect tools allow assessment of the pathogenicity of identified variants using various parameters.

We analyzed 48 unrelated patients with an alleged autosomal dominant familial form of PD using WES technology and developed a comprehensive strategy for data analysis and selection of potential pathogenically meaningful variants using available bioinformatics resources.

## Materials and Methods

### Objects of Research

Studied cohort included 48 unrelated patients with PD. By origin they were all Russians from Moscow and Saint Petersburg. Patients were selected and investigated according to the unified PD rating scale (UPDRS), and Hoehn & Yahr scores (Fahn and Elton, 1987; Goetz et al., 2004) at the Scientific Center of Neurology (Moscow) and Pavlov First Saint Petersburg State Medical University (Saint Petersburg). PD diagnosis was based on the UK PD Bank Criteria (Hughes et al., 1992). The average age at disease onset was 52.6 ± 17.0 (the mean ± standard deviation), sex ratio was 17/31 (M/F). Detailed statistical demographics of the participants is also provided (Supplementary Table S2). Written informed consent was obtained from all participating patients and families according to the Declaration of Helsinki. The study was approved by the Ethics Committee of the Scientific Center of Neurology and Pavlov First Saint Petersburg State Medical University. All patients were pre-typed using the P051-50 (lot C2-0911) and P052-50 (lot C1-0809) probe sets and EK1-FAM reagent kit for the SALSA MLPA MLPR (MRC-Holland b.v., Netherlands) according to the manufacturer’s recommendations (Filatova et al., 2014); all of them did not have any frequent mutations associated with pathogenesis of the PD.

### DNA Preparation and Sequencing

Genomic DNA was obtained from leukocytes using AxyPrepBlood Genomic DNA Miniprep Kit (“Axygen”, USA) as recommended by the manufacturer. The concentration of isolated nucleic acids was measured using Qubit fluorometer (“Invitrogen”, USA) and Quant-iT RNA BR Assay Kit commercial kit as recommended by the manufacturer.

WES was performed using a TruSeq Exome Enrichment kit (Illumina, San Diego, CA, USA) on an Illumina HiSeq 2500 sequencer. Sequence consisted of 201121 exons in 20794 genes with an average coverage of 40×, providing genotype data for 90% of consensus coding exon bases. Resulting potentially pathogenically significant variants in genes listed in Supplementary Table S1 were validated by Sanger sequencing.

### Annotation and Functional Assessment

Bioinformatic analysis of obtained sequences was carried out using the software package “SNP and Variation Suite” (SVS) v.7.4 (Golden Helix, USA). The minor allele frequency (MAF) was estimated using the “1000 genomes” database (1KG^1^), the Exome sequencing project (ESP^2^), Exome Aggregation Consortium Database^3^ and Genome Aggregation Database (gnomAD^4^) using European population as a reference.

The possible pathogenicity of all detected new or rare non-synonymous heterozygous variants was assessed using the dbNSFP v2.9 database (Liu et al., 2013), consisting of SIFT, PROVEAN, Polyphen-2 HVAR and HDIV, FATHMM, MutationAssessor, MutationTaster, LRT, MetaSVM and MetaLR software (Dong et al., 2015), integrated into SVS. Additional annotation was carried out in Combined Annotation Dependent Depletion (CADD; Kircher et al., 2014) and Rare Exome Variant Ensemble Learner (REVEL; Ioannidis et al., 2016). A functional evaluation of selected annotated variants was carried out bioinformatically using the Pathway Studio database (Elsevier, New York City, NY, USA). Only connections based on reliable studies, in which statistically significant results were obtained, were taken into account as “reliable” bonds.

## Results

### Primary Selection of Initial Data and Screening for False-Positive Heterozygotes

**Figure 1:**
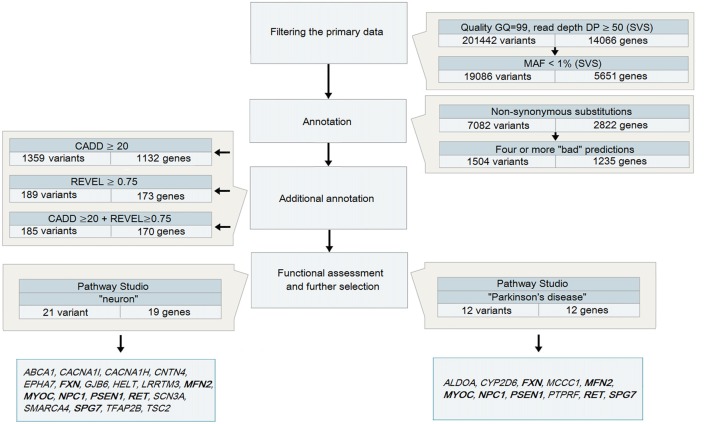
A strategy to analyze data obtained during whole-exome sequencing (WES) for patients with Parkinson’s disease (PD) Note. Genes highlighted in bold are those identified in both strategies.

Variant call format (VCF) files obtained after the analysis of DNA samples from 48 patients with PD were analyzed using SVS: all heterozygous variants (in accordance with the assumed autosomal dominant nature of inheritance) were selected with a genotyping quality GQ > 99 and a coverage of at least 50 reads (Figure 1), because it is believed that variants with high DP are more reliable than those having a low read depth (Bansal, 2010). The total number of such variants for 48 samples was 201,442.

The analysis of heterozygous variants revealed strong differences in the ratio between two allelic variants that were read during the sequencing. The assumption of the presence of false-positive heterozygotes was verified by the Sanger sequencing of individual exons of *LRRK2*. As a result, an approach for the elimination of false-positive heterozygotes was developed at the initial stage of the primary data analysis. The approach was based on the use of the following parameters: DP, i.e., approximated read depth (the number of high-quality mapped reads for the current position), and AD1 and AD2, which were the approximated read depth for the first and the second allele, respectively. Selection criteria were chosen empirically, i.e., heterozygous positions with different read depths and AD1/AD2 ratio were verified using Sanger sequencing (Table 1). As a result, the following criteria were chosen for selecting reliable heterozygous positions: “DP ≥ 50 and |AD1 – AD2|/DP <0.3”. Variants that did not meet the specified criteria were discarded as unreliable. The approach was implemented as a Python (version 2.7) script and is represented in Supplementary Materials.

**Table 1:** Parameters that were used to develop an approach of screening for false-positive heterozygotes.

Location (Grch37)	|AD1 − AD2|/DP	The presence of two alleles (validation with Sanger)
12:40713899	|33 − 42|/75 = 0.12	present
12:40753203	|31 − 21|/52 = 0.23	−/−
12:40758665	|86 − 40|/126 = 0.37	absent
12:40696597	|41 − 13|/52 = 0.54	−/−
12:40748293	|50 − 11|/58 = 0.67	−/−
12:40653331	|76 − 10|/84 = 0.78	−/−

*Note: AD, allelic depth; DP—read depth*.

Further, all new or rare nonsynonymous variants with MAF that did not exceed 1% in 1KG and ESP were selected. As a result, 19,086 heterozygous nonsynonymous variants located in the coding regions of 5651 genes were identified. Two different mutations in one gene were not identified in any of the patients analyzed.

### Annotation

To narrow down the range of candidate variants, we used bioinformatics tools to predict the potential pathogenicity of variants by analyzing both the evolutionary conservation and the conservation in combination with changes in the structural and functional properties of the protein, as well as two tools with combined algorithms. Missense and nonsense variants, which were predicted to be pathogenic by at least four tools, were reserved for further analysis. This step reduced the number of potential variants by almost five times (up to 1504 variants in 1235 genes).

At this stage of the analysis, three pathogenically significant variants in the genes that have been known to be involved in the pathogenesis of PD were identified. All of them are described missense mutations.

To narrow the search even further, additional annotation was conducted using the prediction tools CADD and REVEL, which are neural networks integrating and combining a number of different annotation algorithms that were trained to identify potentially pathogenic variants. We selected the thresholds for the parameters used by the tools based on the objective requirements to the level of rigidity: a CADD score >20 allowed selection of variants belonging to 1% of the most pathogenic variants in the human genome (Kircher et al., 2014), while a REVEL score ≥0.75 provided high specificity for the annotation of rare variants (Ioannidis et al., 2016) and allowed us to narrow the search to 185 variants in 170 genes.

### Functional Assessment

**Figure 2:**
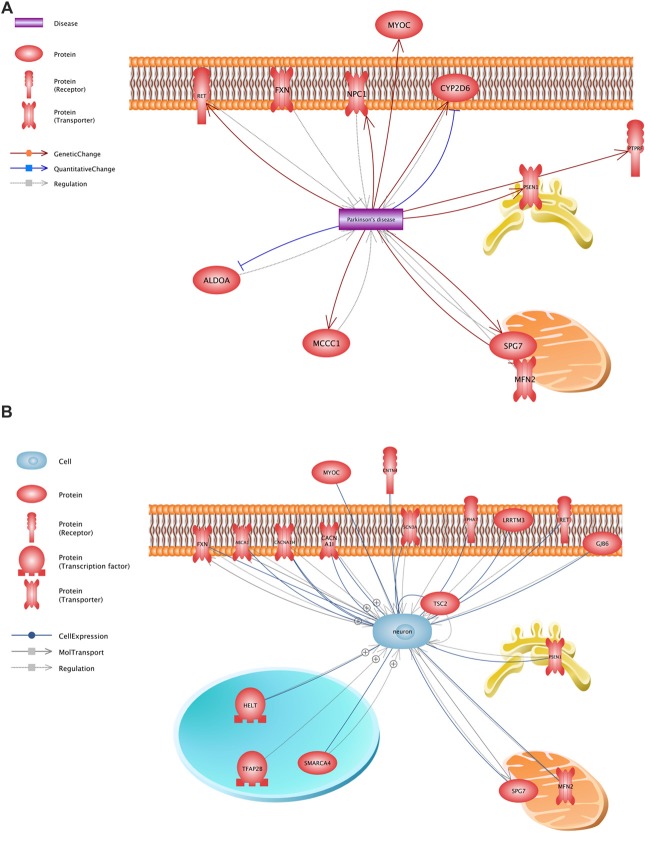
Functional assessment of the proteins encoded by the analyzed genes. **(A)**—a network constructed using the keyword “PD”, **(B)**—a network constructed using the keyword “neuron”.

All variants that were identified on the previous stage were analyzed in Pathway Studio v.11.4 (Elsevier, New York City, NY, USA). Genes were selected using the keywords “PD” and “neuron” (Figure 2). As a result, 23 genes were identified (Figure 1, Supplementary Table S1); seven (*FXN, MFN2, MYOC, NPC1, PSEN1, RET* and *SPG7*) were associated with both keywords.

The presence of potential pathogenically significant variants in all 23 genes was analyzed in 48 patients with PD. They were detected in 21 patients, which was 44% of the total number of PD patients analyzed. Four patients (8%) had two genes with potential pathogenically significant variants. It should be noted that practically only one variant in each gene was identified. Only *SCN3A* had three variants: each variant was found only once in three different patients.

## Discussion

In recent years, increasing numbers of medical genetics studies have been conducted using NGS technology, which is associated with increasing price accessibility and improved tools for *in silico* data analysis. The proportion of genetic factors involved in the pathogenesis of a disease and successfully identified using NGS ranges from 5.6% to 59% to date (Iglesias et al., 2014; Boillot et al., 2015; Izumi et al., 2015). At the same time, researchers found a large number of variants of unknown significance. It is believed that such variants should in no case be excluded from consideration (Wu and Jiang, 2013). As a result, the problem of correct evaluation of their pathogenicity arises. Currently available bioinformatics tools give conflicting predictions of pathogenicity, which greatly complicates the evaluation procedure, especially in the absence of a large family pedigree. The method of prediction is no less important because the decision on the potential pathogenicity is based on a different number of parameters. Tools with differing or combined algorithms in combination with the functional annotation are currently considered the closest to optimal (Wu and Jiang, 2013; Dong et al., 2015).

However, there is currently no optimal approach for selecting potentially pathogenic variants. Thus, an insufficient number of prediction tools is often used (Reis et al., 2013; Vona et al., 2014; Farlow et al., 2016) or no detailed description of the selection strategy is provided (Acke et al., 2014). The optimal approach for selecting and analyzing homozygous variants was proposed in a recent large-scale study devoted to the search for genetic risk factors in patients with early onset PD (Jansen et al., 2017). However, the proposed approach is not suitable for patients with late onset PD, in which heterozygous variants are analyzed. In this regard, we have developed our own multistep approach for the analysis and screening of candidate variants using almost all available bioinformatics resources for the analysis of exonic areas.

The developed approach for the elimination of false-positive heterozygous variants significantly reduces the number of analyzed data, thereby facilitating the work already at the first stages of the study. The tools used to assess the potential pathogenicity of the identified variants are presented in an amount sufficient to compile reliable data on the pathogenicity of analyzed variants because they are based on various predicting strategies, including combinations. CADD, which has been already proven in a similar study (Jansen et al., 2017) in combination with REVEL, which has more stringent evaluation criteria, allows researchers to obtain objective data in an amount sufficient for the subsequent functional analysis. In turn, the Pathway Studio database used for this purpose makes it possible to conduct an accurate and up-to-date functional evaluation.

The developed approach allows selection of only the most pathogenically significant substitutions, strictly excluding all other weakly pathogenic variants that do not fit at least one selection criteria. Thus, when analyzing the data obtained during the WES, none of the three variants identified in the known genes involved in the pathogenesis of PD reached the thresholds (Table 2). This can be explained by the fact that all three variants are most likely weakly pathogenic and considered only as risk factors for the development of PD.

**Table 2:** Variants in genes known to be involved in Parkinson’s disease (PD) pathogenesis.

Gene	dbSNP141 RS ID	Amino acid change	MAF (EXAC)	REVEL score	CADD score	Association with PD	Reference
*GBA*	rs2230288	NM_001171811 E365K	0.0098	0.595	17.33	Risk factor	Benitez et al. (2016)
*GBA*	rs75548401	NM_001171811 T408M	0.0066	0.731	22.2	Risk factor	Benitez et al. (2016)
*LRRK2*	rs34594498	A419V	0.0005	0.175	24.3	Risk factor	Ross et al. (2011) and
							Li et al. (2015)

*Note: MAF, minor allele frequency*.

Thus, the analysis of 48 unrelated patients with an alleged autosomal dominant form of PD allowed us to find a number of genetic factors presumably involved in the pathogenesis of the disease and form a list of 25 potential pathogenically significant variants located in the coding regions of 23 genes. In 22 genes, only one potential pathogenically significant variant was identified, while three variants were detected only in *SCN3A*. Each of these identified variants was found only once in the patients under study. Our findings confirm the recently advanced hypothesis that new genes associated with the pathogenesis of PD appear to include rare single mutations (Jansen et al., 2017). It should be noted that only 7 of 23 genes (*FXN, MFN2, MYOC, NPC1, PSEN1, RET* and *SPG7*) were selected using both keywords. Data of varying significance on the involvement of these genes in the development of the disease are available at present time.

The most convincing evidence of involvement is available for *NPC1* and *PSEN1* genes. For *NPC1*, a hypothesis on the contribution of heterozygous mutations to the risk of PD development was expressed earlier. The hypothesis was built primarily on the basis of information on the comorbidity of Niemann–Pick disease caused by mutations in *NPC1* and parkinsonism (Kluenemann et al., 2013). The situation is the same for the variant p.L173F found in *PSEN1*, it was described as pathogenic in families with Alzheimer’s disease and parkinsonism (Kasuga et al., 2009; Jin et al., 2012). Because the whole symptom complex does not always produce an unambiguous diagnosis and distinguish the underlying disease from those associated with it, the variants we identified can be regarded at least as risk factors for PD development.

Three potential pathogenically significant variants were identified in *SCN3A*, therefore this gene is of importance to PD researchers. The protein encoded by *SCN3A* is a transmembrane sodium channel, which plays an important role in the neuronal function. Disruption of the functioning of such sodium channels can cause the emergence of a number of neurological diseases, including essential tremor (Bergareche et al., 2015), epilepsy (George, 2004; Helbig et al., 2008; Reid et al., 2009; Eijkelkamp et al., 2012; Oliva et al., 2012), multiple sclerosis (Waxman, 2006), painful neuropathy (Faber et al., 2012; Huang et al., 2014), myotonia (Jurkat-Rott et al., 2010; Stunnenberg et al., 2010) and paroxysmal myoplegia (Cannon, 2010). It is interesting to note that all three potentially pathogenetically significant variants have been identified by us for the first time and are located in the hydrophobic fifth segment of the SCHN3A protein that is directly responsible for ion transport. All these observations, although only indirectly thus far, support the possible involvement of *SCN3A* in the process of neurodegeneration.

Thus, the approach we developed to analyze the data obtained in the course of NGS for unrelated patients with an alleged autosomal dominant form of PD allowed effective searching for potential pathogenically meaningful variants, significantly reducing the search range from 7082 to 25 variants. A cohort of a large size would be optimal for the studies conducted with patients without a pronounced family history and without clear large pedigrees. Then one could speak of a clear association between a gene with multiple mutations in it and the development of the disease. In our limited cohort of patients, we observed three patients with different mutations in one gene, which may indicate its involvement in the pathogenesis of the disease. At the same time, these variants have not been identified in the EXAC, 1KG and ESP databases, which also may indicate their possible pathogenetic significance, however this significance has to be proved. The accumulation of the data from different groups of researchers will make it possible to clarify the role of individual genes in the development of the disease. The data obtained can be also verified further using experimental approaches, for example, by studying the mechanism of the influence of a nonsynonymous substitution, with such molecular genetic approaches as RNA interference or modeling using both animal models and cell culture. We realize that the results of the study can handle some false positives since there is the lack of ethnically matching sequenced Russian (or at least Slavonic) control population that can be used as the reference dataset for such type of analysis. Besides, the comparison of different datasets is also complicated by a well-known low concordance of differing sequencing platforms and pipelines used for variant calling (O’Rawe et al., 2013). Nevertheless, our results first of all provide some valuable data that will be further accumulated and estimated by the scientific community worldwide.

## Data Availability

The raw data supporting the conclusions of this manuscript will be made available by the authors, without undue reservation, to any qualified researcher.

## Author Contributions

MS performed next-generation sequencing data analysis, sanger sequencing studies and wrote the manuscript. EF, NA and AY contributed to the collection of the clinical data and its analysis. AA and TU carried out the genotyping of patients with PD. IV and AE performed the primary bioinformatics analysis of the next-generation sequencing data. MS and PS designed and coordinated the study and wrote the manuscript. SI and SP were involved in revising the manuscript critically for important intellectual content. VZ and AE performed the primary bioinformatics analysis of the next-generation sequencing data, IV created the final appropriate version of the Python script. All authors read and approved the final manuscript.

## Conflict of Interest Statement

The authors declare that the research was conducted in the absence of any commercial or financial relationships that could be construed as a potential conflict of interest.

## Footnotes

^1^http://www.1000genomes.org

^2^https://esp.gs.washington.edu/drupal/

^3^http://exac.broadinstitute.org/

^4^http://gnomad.broadinstitute.org/
