# Supplementary material for: Whole-Exome Sequencing in Searching for New Variants Associated With the Development of Parkinson’s Disease
Source: Front Aging Neurosci. 2018 May 15;10:136. doi: 10.3389/fnagi.2018.00136 (PMC5963122; doi:10.3389/fnagi.2018.00136)
Supplement: Supplementary file 1 [file Data_Sheet_1.DOCX]

Supplementary Material

Whole-exome Sequencing in Searching for New Variants Associated with the Development of Parkinson’s Disease

Marina V. Shulskaya^*^, Vladimir V. Zyrin, Anelya Kh. Alieva, Ekaterina Yu. Fedotova, Natalia Yu. Abramycheva, Tatiana S. Usenko, Andrei F. Yakimovsky, Anton K. Emelyanov, Sofya N. Pchelina, Sergei N. Illarioshkin, Petr A. Slominsky, and Maria I. Shadrina

*** Correspondence:** Marina V. Shulskaya: m.shulskaya@gmail.com

# Supplementary Data

**The algorithm for selecting reliable heterozygous variants presented in the form of a Python (version 2.7) script**

#In this script we select the high-confidence heterozigous variants from vcf file, according to the criteria described in th paper.

# Using octotorp/exclamation mark we select the correct interpretator for following code

#! /usr/bin/python

import re #we import the module for working with regular expressions

fw = open('result.vcf', 'w') # we are opening result.vcf file to write the output lines there

with open('./source.vcf') as f: # we are opening source.vcf as an input file

for line in f: #iterating upon every line in vcf file

if line.find("0/1") > 1: # we select only the lines with at least one instance of "0/1" string, which are accordingly to VCF 4.2 documentation are heterozigous variants

a = line.split() #we are splitting the line with variant by the whitespaces (whitespace is a space, tabulation, etc.. Standart delimiter betwen columns in vcf format file is tabulation)

b = re.split(':|,', a[len(a)-1]) # we split the the INFO column by it's own ":" or "," delimiter,to extract Genotype Quality(GQ), read depth (DP) and Allele Depth(AD, one for each allele) for this variant.

if (int(b[4]) == 99 and int(b[3]) >= 50 and abs(float(b[1])-float(b[2]))/float(b[3]) < 0.3): # If Genotype Quality is >99, read depth(DP) is at least 50, and module of (depth of the first allele - depth of the second allele)/DP < 0.3 we are selecting this variant to be in the output. Otherwise we are interating upon next line of vcf

fw.write(line) #writing the line to the output file, if formerly described conditions are met

fw.close() #closing the link to the output file
