# Supplementary material for: Whole-Exome Sequencing in Searching for New Variants Associated With the Development of Parkinson’s Disease
Source: Front Aging Neurosci. 2018 May 15;10:136. doi: 10.3389/fnagi.2018.00136 (PMC5963122; doi:10.3389/fnagi.2018.00136)
Supplement: Supplementary file 2 [file Data_Sheet_2.docx]

**Table 1S.** The list of potential pathogenetically significant variants identified in patients with an alleged autosomal dominant familial form of PD

| **Gene** | **dbSNP141 RS ID** | **Amino acid substitution** | **MAF (EXAC)** | **REVEL score** | **CADD score** |
| --- | --- | --- | --- | --- | --- |
| *ABCA1* | - | NM_005502 p.Q732R | - | 0.88 | 26.0 |
| *ALDOA* | rs760807537 | NM_001127617 p.H220R | 1.499e-05 | 0.93 | 25.0 |
| *CACNA1H* | rs770552911 | NM_001005407 p.T1609P | - | 0.87 | 25.0 |
| *CACNA1I* | rs376169293 | NM_001003406 p.G1196S | 0.0003 | 0.86 | 33.0 |
| *CNTN4* | rs200709524 | NM_001206955 p.V938G | - | 0.81 | 24.0 |
| *CYP2D6* | rs1058172 | NM_000106 p.R365P | 1.522e-05 | 0.98 | 35.0 |
| *EPHA7* | - | NM_004440 p.V887G | - | 0.79 | 28.0 |
| *FXN* | rs760965156 | NM_000144 p.D139Y | 1.649e-05 | 0.82 | 31.0 |
| *GJB6* | - | NM_001110219 p.L6P | - | 0.96 | 23.1 |
| *HELT* | - | NM_001300781 p.R63G | - | 0.87 | 28.6 |
| *LRRTM3* | - | NM_178011 p.N119H | - | 0.83 | 24.0 |
| *MCCC1* | - | NM_001293273 p.G94R | - | 0.92 | 34.0 |
| *MFN2* | rs119103267 | NM_001127660 p.R707W | 0.0006 | 0.84 | 35.0 |
| *MYOC* | rs754237376 | NM_000261 p.A427T | - | 0.87 | 29.0 |
| *NPC1* | rs150334966 | NM_000271 p.S1004L | 0.0015 | 0.76 | 24.1 |
| *PSEN1* | - | NM_000021 p.L173F | - | 0.92 | 25.1 |
| *PTPRF* | - | NM_130440 p.R1575Q | - | 0.84 | 34.0 |
| *RET* | rs148935214 | NM_020630 p.S649L | 0.0005 | 0.76 | 25.1 |
| *SCN3A* | - | NM_006922 p.D1227G | - | 0.93 | 29.1 |
| *SCN3A* | - | NM_006922 p.D1227E | - | 0.86 | 25.0 |
| *SCN3A* | - | NM_006922 p.N1290H | - | 0.76 | 24.1 |
| *SMARCA4* | rs201128299 | NM_001128844 p.V766G | 0.0036 | 0.97 | 29.1 |
| *SPG7* | rs61755320 | NM_003119 p.A510V | 0.0040 | 0.92 | 32.0 |
| *TFAP2B* | - | NM_003221 p.I291N | - | 0.96 | 31.0 |
| *TSC2* | - | NM_000548 p.F1606L | - | 0.86 | 26.4 |

Note: MAF - minor allele frequency.

**Table 2S.** Detailed statistical demographics of the participants

| **Accession No.** | **City** | **Nationality** | **Age** | **Sex** |
| --- | --- | --- | --- | --- |
| 1 | Moscow | slavonic | 61 | m |
| 2 | Moscow | slavonic | 56 | m |
| 24 | Moscow | slavonic | 45 | f |
| 74 | Moscow | slavonic | 67 | f |
| 82 | Moscow | slavonic | 61 | m |
| 84 | Moscow | slavonic | 47 | f |
| 89 | Moscow | slavonic | 51 | m |
| 101 | Moscow | slavonic | 47 | f |
| 112 | Moscow | slavonic | 68 | f |
| 129 | Moscow | slavonic | 56 | m |
| 158 | Moscow | slavonic | 45 | m |
| 165 | Moscow | slavonic | 53 | f |
| 167 | Moscow | slavonic | 43 | m |
| 172 | Moscow | slavonic | 56 | m |
| 175 | Moscow | slavonic | 41 | f |
| 319 | Moscow | slavonic | 66 | f |
| 338 | Moscow | slavonic | 47 | m |
| 339 | Moscow | slavonic | 39 | f |
| 342 | Moscow | slavonic | 64 | f |
| 346 | Moscow | slavonic | 69 | f |
| 347 | Moscow | slavonic | 37 | m |
| 351 | Moscow | slavonic | 60 | m |
| 391 | Moscow | slavonic | 56 | f |
| 396 | Moscow | slavonic | 41 | m |
| 400 | Moscow | slavonic | 55 | f |
| 401 | Moscow | slavonic | 57 | f |
| 407 | Moscow | slavonic | 49 | f |
| 417 | Moscow | slavonic | 54 | m |
| 427 | Moscow | slavonic | 35 | f |
| 10m | Moscow | slavonic | 40 | f |
| pd87 | Saint-Petersburg | slavonic | 54 | m |
| pd94 | Saint-Petersburg | slavonic | 51 | m |
| pd98 | Saint-Petersburg | slavonic | 81 | f |
| pd100 | Saint-Petersburg | slavonic | 67 | f |
| pd105 | Saint-Petersburg | slavonic | 53 | f |
| pd122 | Saint-Petersburg | slavonic | 60 | f |
| pd129 | Saint-Petersburg | slavonic | 59 | f |
| pda1 | Saint-Petersburg | slavonic | 73 | f |
| pda2 | Saint-Petersburg | slavonic | 69 | f |
| pda35 | Saint-Petersburg | slavonic | 70 | f |
| pda37 | Saint-Petersburg | slavonic | 67 | f |
| pdm9 | Saint-Petersburg | slavonic | 56 | f |
| pdm20 | Saint-Petersburg | slavonic | 65 | f |
| pdm25 | Saint-Petersburg | slavonic | 82 | f |
| pdm26 | Saint-Petersburg | slavonic | 61 | m |
| pdm32 | Saint-Petersburg | slavonic | 59 | f |
| pdm39 | Saint-Petersburg | slavonic | 66 | m |
